# Distribution Characteristics and Adaptation Mechanisms of Exotic Spontaneous Plant Diversity in Urban Road Green Spaces of Changchun, China

**DOI:** 10.3390/plants15010107

**Published:** 2025-12-30

**Authors:** Diyang Liu, Congcong Zhao, Yongfang Wang, Yuandong Hu

**Affiliations:** 1 College of Landscape Architecture, Northeast Forestry University, Harbin 150040, China; 2022120657@nefu.edu.cn (D.L.); zhao_congcong@nefu.edu.cn (C.Z.); wangyongfang2000@163.com (Y.W.); 2 XAUAT-UWA International Joint Lab on Urban Biodiversity and Design, Xi’an University of Architecture and Technology, Xi’an 710055, China

**Keywords:** urban road green spaces, spontaneous plant diversity, exotic plants, distribution patterns, adaptation mechanisms

## Abstract

Spontaneous plants are plants that occur in urban environments such as pavement gaps or cracks in walls without cultivation and are not remnants of historic native habitats. They are critical components of urban road green space vegetation, and their distribution is affected by multiple factors. Heavy traffic and frequent human disturbances on urban roads exacerbate exotic spontaneous plant invasions. Exploring the diversity of their distributions, adaptation mechanisms of these exotic plants and their relationship with native ones is vital for focused control of harmful invasives. Based on field surveys, this study analyzed the distribution of exotic spontaneous plants across habitat types, urbanization gradients and disturbance intensities in road green spaces, and their interactions with native counterparts. Our results indicated: (1) 425 spontaneous species were recorded (234 exotic, 191 native), with 71.8% cosmopolitan and 74.7% monotypic genera. (2) The spontaneous exotic plant community achieves extensive resource preemption by forming a structure dominated by a single super-dominant species (Setaria viridis) and characterized by a broader overall niche breadth. (3) Different habitats sustain a similar number of exotic spontaneous plant species (i.e., α-diversity), but their species compositions are highly differentiated, with such differences driven almost entirely by species turnover. At the urban scale, spontaneous exotic plants adapt to regional environments with varying urbanization intensities by maintaining extensive similarity in community composition and making only extremely weak adjustments to the pattern of individual distribution among species. (4) The spontaneous plant community exhibits a pattern dominated by weak interspecific associations and random assemblages, where ecological interactions among species are weak, and the community structure is more consistent with the stochastic processes described by the Neutral Theory. At the regional environmental gradient scale, the diversity of spontaneous native and exotic plants exhibited coordinated variation. The study provides a scientific basis for urban biological invasion control and biodiversity management.

## 1. Introduction

Biological invasion can be summarized as a multi-stage dynamic process in which a species crosses geographical barriers through human activities to enter a new area and then sequentially overcomes multiple obstacles such as environmental constraints, reproductive challenges, and dispersal limitations, establishes populations, and ultimately exerts negative impacts on the local ecology, economy, and other aspects [1]. According to their spread status, they can be classified into naturalized plants, invasive plants, and escaped cultivated plants. They represent different states in the process of “introduction → escape (escaped species) → population establishment (naturalized species) → widespread spread (invasive species)” [2,3]. Driven by globalization, specific pathways such as international trade, horticultural introduction, and transportation networks have significantly increased the risk of exotic species spread. Meanwhile, climate change may further exacerbate this threat—for instance, rendering certain drought-adapted species that have reached their physiological tolerance limits more vulnerable. In recent years, scientific research has been improving the precision of risk management and control through data integration and mechanistic modeling. For example, distinguishing between the “invasion” and “expansion” processes of pine wood nematode disease and quantifying the contributions of anthropogenic and natural dispersal can provide direct evidence for regionalized prevention and control strategies; systematically integrating citizen science and traditional survey data can more comprehensively reveal species niches, thereby enhancing the reliability of large-scale risk prediction [4,5].

China attaches great importance to the issue of exotic species invasion. The National Strategy and Action Plan for Biodiversity Conservation (2011–2030), issued in 2010, incorporated the prevention and control of invasive alien species into the core goals of national biodiversity conservation. However, restricted by multiple objective factors, China remains one of the countries most threatened by biological invasions and suffering the most severe losses globally [6,7]. According to the Report on the State of the Ecology and Environment in China 2020, more than 660 invasive alien species have been recorded in China, ranking among the highest in the world; among the 100 most threatening invasive alien species globally listed by the International Union for Conservation of Nature (IUCN), 50 are present in China. These species cause direct economic losses exceeding 200 billion yuan annually, placing China in a grim situation regarding the prevention and control of invasive exotic species [8,9].

A large number of studies on urban exotic spontaneous plants have been conducted domestically and internationally, mostly focusing on aspects such as their diversity distribution patterns [10,11], driving factors (e.g., land use, transportation networks, socioeconomic factors) [12,13,14,15,16,17,18,19,20,21,22,23], and impacts on the diversity of native spontaneous plants. Foreign research on exotic spontaneous plants started earlier, and many hypotheses on biological invasions have been proposed, such as the “Darwin’s Naturalization Hypothesis” and the “Adaptation Hypothesis” [24]. Compared with domestic research, foreign studies focus more on invasion causes [25,26] and invasion mechanisms [27]. In contrast, domestic research on interspecific relationships is mostly concentrated in forests, riparian zones, and other areas [28,29,30], with fewer studies on highly urbanized habitats. Existing studies have shown that, in addition to environmental influences, the interactions between exotic spontaneous plants and native spontaneous plants are also one of the factors affecting the naturalization or invasion of exotic plants [13,31].

However, most domestic studies have been carried out in southern provinces/cities or megacities (such as Guangdong Province and Beijing), with relatively insufficient attention paid to cities in northern temperate or frigid-temperate zones, especially those in the old industrial base of Northeast China [32]. Furthermore, there is a paucity of in-depth research on urban spatial heterogeneity, spontaneous plants, ecological processes, and the mechanisms of human disturbance in these regions. Existing research on exotic spontaneous plants in cold regions also mostly focuses on farmlands and forests, neglecting road green spaces as a “key corridor,” resulting in a lack of scientific basis for the prediction and prevention of invasion risks in cold-region cities [33]. Combined with the habitat fragmentation effect of high-density road networks [34], and the superimposed impacts of human activities [35], road green spaces have the potential to become suitable habitats for exotic spontaneous plants. Therefore, studying the distribution patterns of exotic spontaneous plants in road green spaces is of great significance.

Spontaneous plants refer to plants that are not intentionally introduced or cultivated, and they establish and regenerate naturally through their own propagules. They have high sensitivity and wide adaptability, can quickly respond to urban environments, and are excellent research objects. Changchun City, a regional central city in Northeast Asia, has a well-developed transportation network. As a northern cold-region city, the spontaneous plants in its road green spaces are affected by the superposition of urbanization and the special habitats of cold regions, which may form unique distribution patterns and adaptation strategies [36,37]. However, existing studies have not specifically covered this area.

In summary, studying the distribution of exotic spontaneous plants and their interaction mechanisms with native spontaneous plants in the road green spaces of Changchun City is of great significance for the management of exotic plants and is also an important part of biodiversity conservation. Therefore, based on a series of scientific questions, such as the diversity distribution patterns of exotic spontaneous plants, how they adapt to the highly heterogeneous urban habitats, and their diversity interactions and interspecific relationships with native spontaneous plants, this study takes the spontaneous plants in the road green spaces of Changchun City as the research object and aims, through quadrat surveys, to: (1) reveal the distribution patterns of exotic spontaneous plants; (2) clarify their strategies for adapting to heterogeneous urban habitats; (3) explore their interspecific relationships with native spontaneous plants. The research results will provide a scientific basis for the precise management and diversity conservation of plants in cold-region cities.

To this end, we propose the following hypotheses: (1) The richness of exotic plants exhibits a regular variation along the environmental gradient from urban centers to rural fringes—characterized by a gradual decrease in the intensity of human disturbance—and is expected to show significant dominance in habitats subjected to high-intensity human disturbance; (2) Successfully established exotic plants adapt to urban habitats by exhibiting a suite of functional traits conducive to dispersal, rapid growth, and stress tolerance, and their ecological niche breadth is wider than that of native plants, enabling them to exploit a broad range of urban disturbed resources; (3) Based on the Biotic Resistance Hypothesis, the abundance of exotic plants is predicted to be suppressed in communities with high native plant diversity, and the interspecific association network between exotic and native plants is hypothesized to be dominated by competitive interactions.

## 2. Results

### 2.1. Characteristics of Species Diversity Composition of Exotic Spontaneous Plants

#### 2.1.1. Analysis of Family-Genus Composition and Geographical Elements

**Figure 1:**
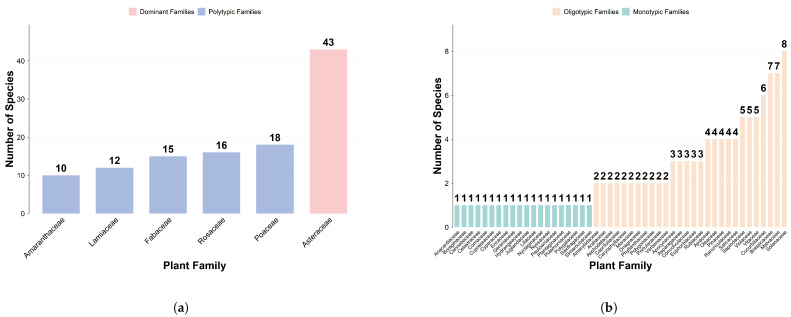
Family Composition of Exotic Spontaneous Plants. (**a**) Number of species in dominant families and diverse families. (**b**) Number of species in oligotypic families and monotypic families.

A total of 54 families, 169 genera, and 234 species of exotic spontaneous plants were recorded in this floristic survey (Figure 1). The number of families is abundant (54 families), among which Asteraceae is the absolute dominant family, accounting for 18% of the total number of exotic spontaneous plant species. Poaceae, Rosaceae, Fabaceae, and other families are the main component families of exotic plants; oligotypic families and monotypic families together consist of 48 families, contributing 50% of the total number of species. This indicates that exotic spontaneous plants are scattered in distribution at the family level, and have strong diffusion and adaptability, with diverse family origins and multiple invasion pathways.

Monotypic genera account for 74.7% of the total genera and contribute 54% of the total species, whereas polytypic genera (e.g., tetratypic and pentatypic genera) only account for 2.4% and 1.2% of the total genera, respectively (Table 1). The predominance of monotypic genera coupled with a severe lack of dominant genera results in an extremely scattered distribution of species at the generic level in the study area. This pattern may be jointly driven by continuous horticultural introduction, strong anthropogenic selection preferences, and the climatic filtering effect of the urban environment. These factors induce sustained and diversified propagule pressure, which enables a large number of genera to be successfully established and maintain populations with only a few individual species, thereby inhibiting the formation of a small number of dominant genera. This result is consistent with the global general pattern that urban floras tend to be internationalized and homogenized [38]: urban environments filter and assemble plant species from all over the world, yet typically only a small number of widely adaptable species can become dominant, while most species exist in a low-frequency and isolated manner. In addition, the evolutionary history of the flora in the study area (e.g., multiple independent immigration events) and local random colonization processes may also play a certain role in shaping this pattern.

**Figure 2:**
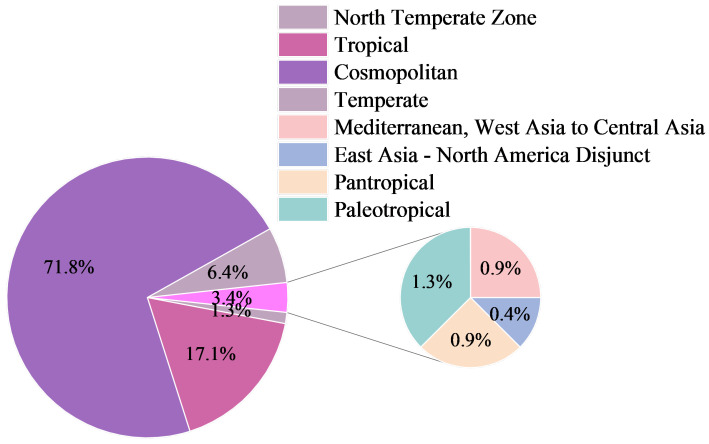
Floristic Composition of Exotic Spontaneous Plants.

In terms of geographical elements (Figure 2), cosmopolitan species account for 71.8% of the total number of species, tropical elements account for 17.1%, and temperate elements (mainly North Temperate Zone distribution, etc.) account for 6.4%. Elements such as the Mediterranean-Central Asia distribution type, pantropical distribution type, and East Asia-North America disjunct distribution type account for 2.2%. Exotic spontaneous plants are dominated by highly adaptable cosmopolitan species, which reflects that, under the influence of intense human disturbance and globalization, cosmopolitan species have strong environmental tolerance and are easy to establish communities in new habitats. The relatively large number of tropical elements indicates that road greening in Changchun tends to plant tropical plants, such as *Phyla nodiflora* (L.) Greene and *Mirabilis jalapa* L.

#### 2.1.2. Composition Characteristics of Dominant Species and High-Frequency Species

**Figure 3:**
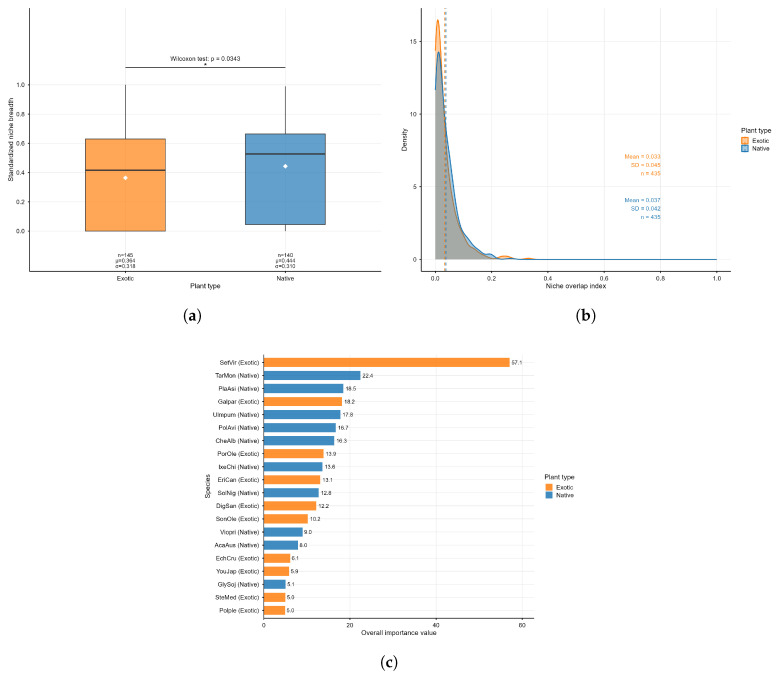
A Comparative Analysis of Ecological Niches and Importance between Spontaneous Native and Exotic Plants (**a**) Boxplot of Niche Breadth for Spontaneous Native and Exotic Plant Species. (**b**) Distribution of Niche Overlap Between Spontaneous Native and Exotic Plant Species. (**c**) Comparative Bar Chart of the Top 10 Species Ranked by Importance Value Among Spontaneous Native and Exotic Plants.

The results of this study indicate that there are fundamental differences in niche structure between spontaneous native plant communities and exotic plant communities. The spontaneous exotic plant community is dominated by a single super-dominant species—*Setaria viridis* (abbreviated as SetVir)—with a comprehensive importance value (IV) as high as 57.05, which is 2.5 times that of *Taraxacum mongolicum* (IV = 22.43), the dominant species of spontaneous native plants (Figure 3c). At the community level, the niche breadth of spontaneous exotic plants is significantly wider than that of spontaneous native plants (Wilcoxon test, p<0.05; Figure 3a). The null model test confirmed that a higher proportion of spontaneous exotic species (17.2%) exhibit niche breadth significantly higher than the random expectation, compared with spontaneous native species (14.3%).

In addition, although the overall proportion of species pairs with significant niche overlap is similar between the two types of plant communities, the niche overlap intensity among dominant spontaneous exotic species is extremely high. The observed maximum niche overlap occurs between the dominant species *Setaria viridis* and *Galinsoga parviflora*, with an overlap index reaching 0.330 (Figure 3b). In contrast, the niche overlap network of dominant spontaneous native species exhibits a more moderate and dispersed pattern.

### 2.2. Distribution Pattern of Exotic Spontaneous Plant Diversity

#### 2.2.1. Diversity Distribution Characteristics of Exotic Spontaneous Plants in Different Habitats

**Figure 4:**
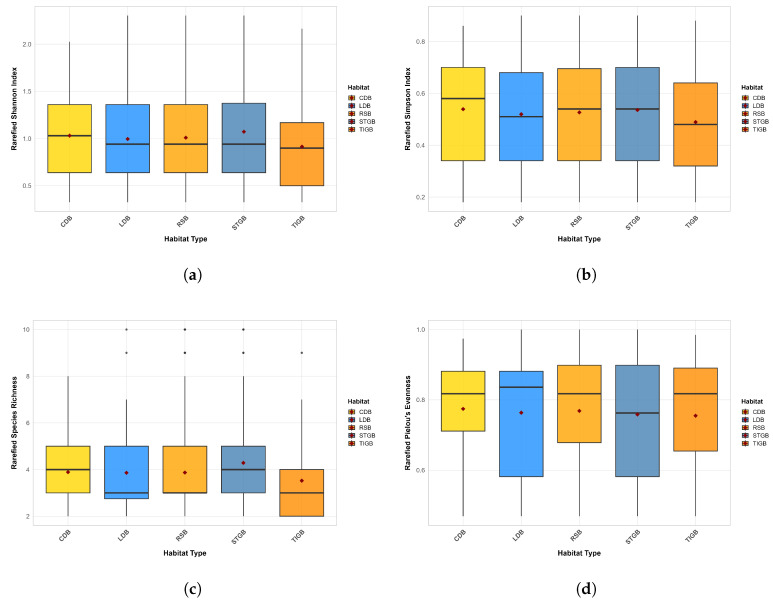
Correlation Analysis between Different Habitat Types and Exotic Spontaneous Plant Diversity. (**a**) Shannon–Wiener Index. (**b**) Simpson’s Diversity Index. (**c**) Species Richness. (**d**) Pielou’s Evenness Index.

Based on the Kruskal–Wallis nonparametric test for the diversity of exotic spontaneous plants across different habitats (Figure 4), the α-diversity of exotic spontaneous plants showed no significant differences among different habitats (all p>0.05), with an extremely small effect size ($\varepsilon^{2}<0.01$). This result was consistent between the observed data and the rarefaction-corrected data.

This indicates that each habitat supports a similar level of species diversity. This does not, however, indicate identical species composition; instead, there may be a scenario of “equal diversity but distinct species composition” across different habitats.

**Figure 5:**
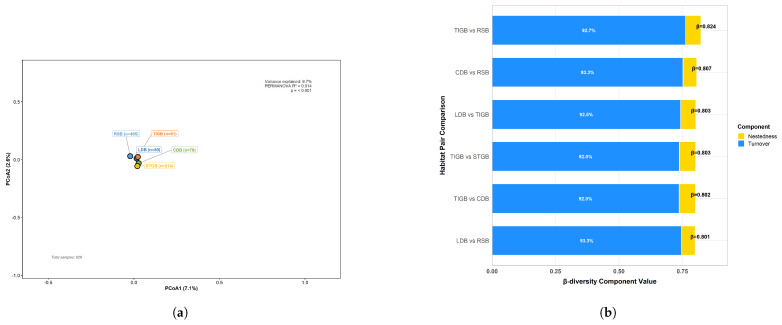
β-Diversity and Partitioning of Exotic Spontaneous Plants Across Habitat Types. (**a**) PCoA Ordination Plot Without Ellipses. (**b**) Stacked Bar Chart of β-Diversity Partitioning.

Non-metric Principal Coordinates Analysis (PCoA) analysis based on Bray–Curtis distances was conducted, and the results of PERMANOVA analysis revealed that $R^{2}=0.014$, F=2.931, and p=0.001 (Figure 5). To explore the components underlying such differences, we performed a β-diversity partitioning analysis. The Sørensen index indicated that the overall β-diversity reached 0.997, among which species turnover contributed 99.9%, while species nestedness accounted for only 0.1%. In habitats with high overall similarity, any detectable limited differences are driven almost entirely by species turnover.

#### 2.2.2. Diversity Distribution Characteristics of Exotic Spontaneous Plants Across Different Urbanization Levels

**Figure 6:**
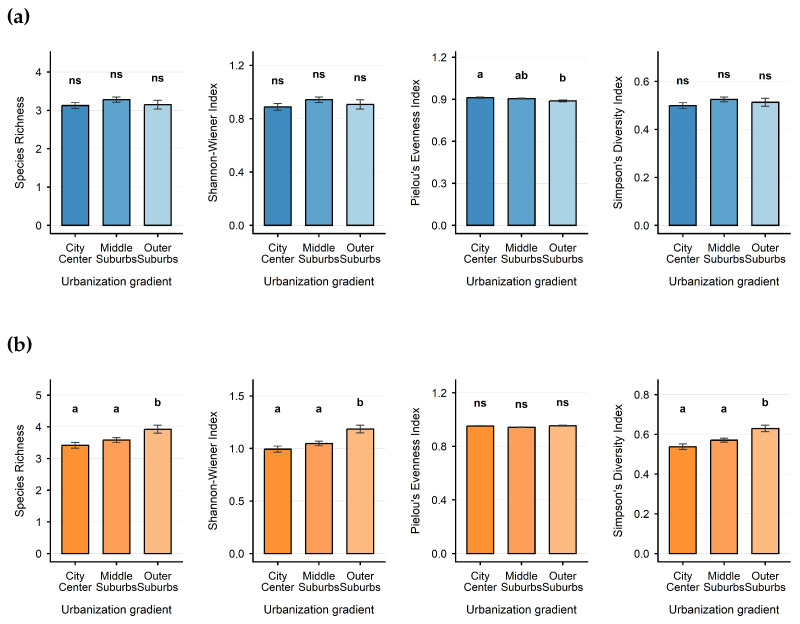
Effects of Disturbance Intensities on the Diversity Distribution Patterns of Native Spontaneous and Exotic Spontaneous Plants. (**a**) α diversity of spontaneous exotic plants along different urbanization gradients. (**b**) α-diversity of spontaneous native plants along different urbanization gradients. Different letters indicate significant differences (Tukey’s HSD test, *p* < 0.05). “ns” = not significant. Error bars = SEM.

For plant occurrence frequency, a binomial generalized linear model (GLM) was used to test the differences in α-diversity between exotic spontaneous and native plants across different habitat types (Figure 6).

Despite similarly weak effect sizes ($\varepsilon^{2}$ = 0.002–0.013), alpha diversity analysis revealed the following statistically identifiable spatial structures that may have limited practical implications: For spontaneous exotic plants, their Pielou’s evenness exhibited a significant statistical gradient from the urban center (a) to the inner suburbs (ab) and further to the outer suburbs (b). This suggests that the evenness of individual distribution among exotic species may undergo a systematic yet extremely weak change along the urbanization pressure gradient. But for spontaneous native plants, their species richness, Shannon index, and Simpson index, the diversity levels in the urban center and inner suburbs were statistically similar (Group a), and both were significantly higher than those in the outer suburbs (Group b). This implies that the urban core and peripheral areas may represent two statistically distinguishable yet practically undifferentiated states in terms of maintaining native plant diversity.

**Figure 7:**
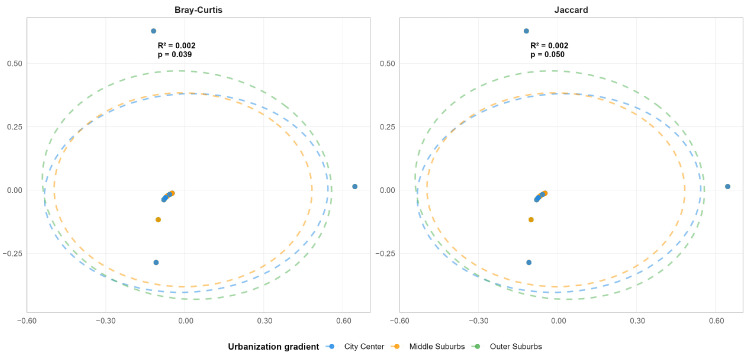
PCoA Confidence Ellipses of β-Diversity for Spontaneous Plants Along Urbanization Gradients.

As can be seen from (Figure 7), Principal Coordinates Analysis (PCoA) showed that there were no extremely significant statistical differences in plant community composition among regions (*p* > 0.05). Meanwhile, the extremely low effect size ($\eta^{2}=0.002$) indicated that “region type” had a limited contribution to explaining the variation in overall community composition and failed to form a clear, stable spatial gradient or grouping pattern.

Despite the a priori assumption of environmental filtering effects potentially induced by the urban gradient, the data analysis did not support the occurrence of systematic changes in plant community composition along the urban center-to-outer suburb gradient. The extremely low explanatory variance suggests that, compared with macro-scale urban gradient zoning, other local environmental factors or stochastic processes may play a more dominant role in the assembly of plant communities within the study area.

### 2.3. Interspecific Relationships Between Exotic Spontaneous Plants and Native Spontaneous Plants

Analysis of the overall interspecific association of species spontaneous herbaceous plants showed that the variance ratio (VR) was 1.4557, which is greater than 1, indicating a positive association among dominant species. The test statistic *W* was 1844.363. At the 0.05 significance level, the upper critical value of the chi-square distribution was $\chi_{0.95}^{2}=1350.921$, and the lower critical value was $\chi_{0.05}^{2}=1185.352$. Since the *W* statistic fell within the right-tailed rejection region, there was an extremely significant overall positive association among the dominant spontaneous species in the road green spaces of Changchun City.

**Figure 8:**
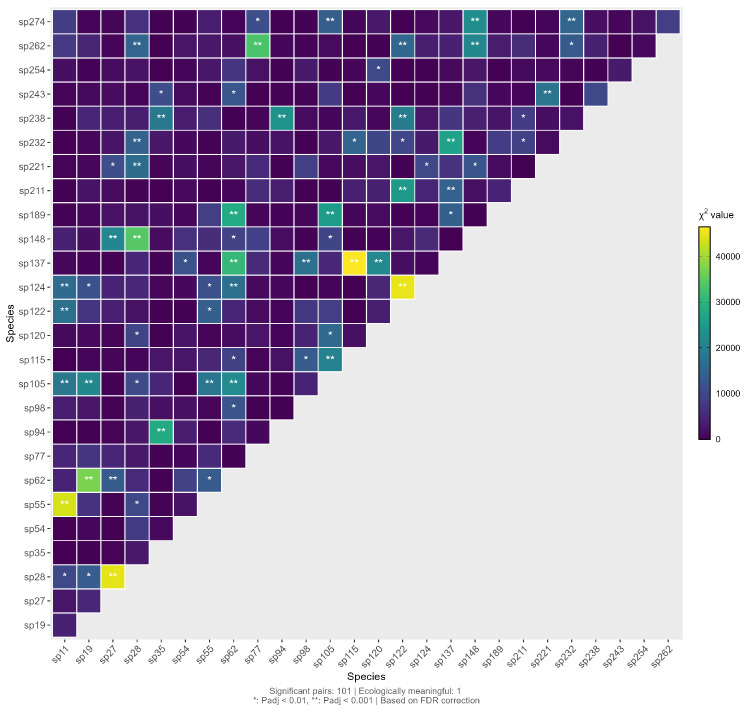
Semi-matrix Plot of $\chi^{2}$ Test for Interspecific Association of Dominant Spontaneous Herbaceous Plant Species. For details on species, see the Appendix A Table A1.

As shown in (Figure 8), we analyzed the 351 species pairs among the 27 dominant species using Fisher’s exact test combined with FDR multiple comparison correction. The results showed that, after FDR correction, a total of 67 species pairs (19.09%) exhibited significant associations (corrected p<0.05). Further analysis incorporating effect size criteria (|V|>0.3, |AC|>0.3) revealed only 1 species pair (0.28%), sp27–sp28, with a statistically significant positive association that also had ecological relevance; no ecologically meaningful negative associations were detected. These findings indicate that, although certain statistical associations exist among the dominant spontaneous species in the road green spaces of Changchun City, there are relatively few interspecific associations with strong ecological significance.

**Figure 9:**
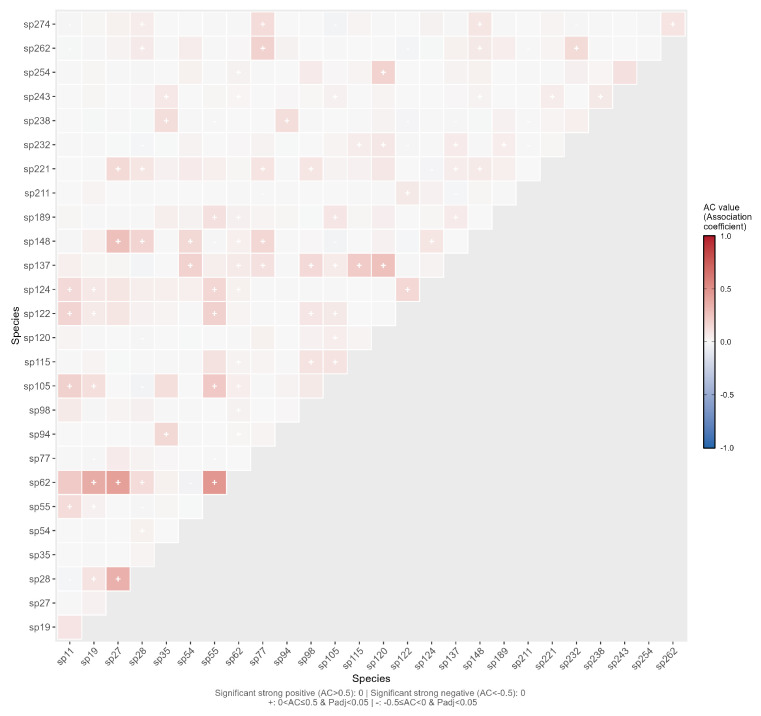
Semi-matrix Plot of AC Values for Interspecific Association of Dominant Spontaneous Plant Species.

In Figure 9, there were **0 pairs** with strong positive association (AC>0.5), **196 pairs (55.84%)** with positive association (0<AC≤0.5), **155 pairs (44.16%)** with negative association (−0.5≤AC<0), and **0 pairs** with strong negative association (AC<−0.5).

**Figure 10:**
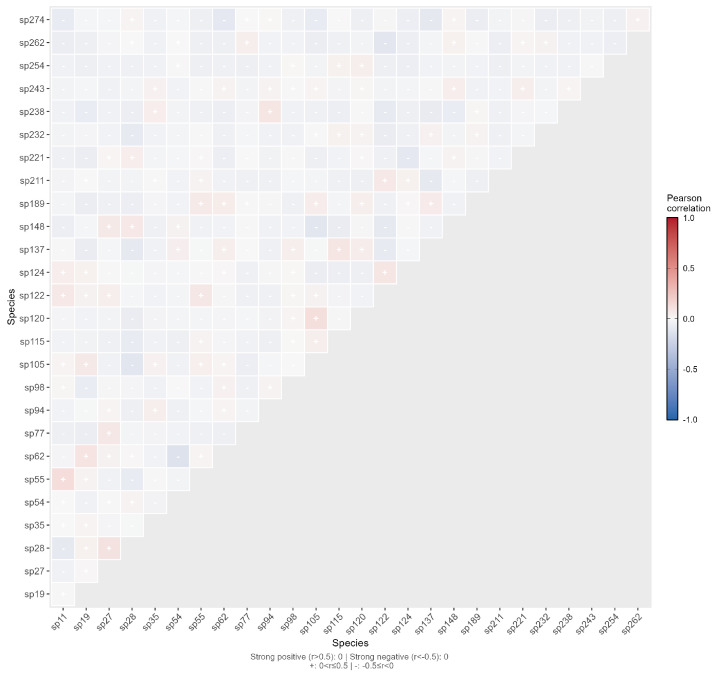
Semi-matrix Plot of Pearson’s Rank Correlation Test for Interspecific Association of Dominant Spontaneous Plant Species.

As shown in (Figure 10), the median of the Pearson correlation coefficients was **−0.0232** and the mean was **−0.0173**, indicating a weak negative correlation overall. The absolute values of the correlation coefficients for all 351 species pairs were less than 0.3, meaning **100% of the pairs exhibited weak correlations** (|r|≤0.3). Among these pairs, 100 (28.49%) showed positive correlations, while 251 (71.51%) showed negative correlations, with negative correlations being dominant.

**Figure 11:**
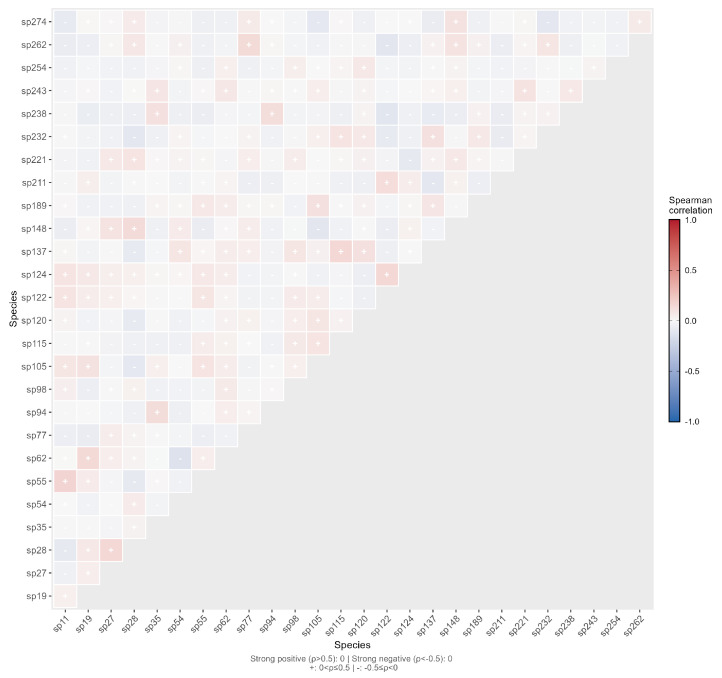
Semi-matrix Plot of Spearman’s Rank Correlation Test for Interspecific Association of Dominant Spontaneous Plant Species.

The median of the Spearman correlation coefficients was −0.0017, and the mean was 0.0036, indicating that the overall correlation was close to 0 but slightly positive. Similarly, the absolute values of the Spearman correlation coefficients for all species pairs were less than 0.3, meaning 100% of the pairs showed weak correlations. Among these pairs, 169 (48.15%) exhibited positive correlations, and 182 (51.85%) showed negative correlations, with positive and negative correlations being nearly balanced (Figure 11). The correlation coefficient analysis revealed that the interactions among dominant spontaneous plants in the road green spaces of Changchun City were dominated by weak correlations (|r| < 0.3 for 78.35% of species pairs). This proportion was significantly higher than that in natural forest ecosystems (typically 30–50%), reflecting the uniqueness of species interactions in urban artificial ecosystems. Strong correlations were extremely rare (|r| > 0.5 for only 5.98% of species pairs) and were predominantly positive (with a positive-to-negative ratio of 20:1), indicating that coordinated changes existed but were limited in intensity. The Spearman correlation coefficients were highly consistent with the Pearson results (r = 0.97), which verified the robustness of the analysis outcomes. This pattern of weak interspecific associations may be attributed to the high heterogeneity of urban environments, frequent anthropogenic disturbances, and the fact that most spontaneous plants are r-strategist species.

### 2.4. Interaction Relationships of Diversity Between Exotic Spontaneous Plants and Native Spontaneous Plants

This study systematically analyzed the relationship between spontaneous native and alien plant diversity across 1267 quadrats distributed in five habitat types using multi-model inference (based on AICc) and Bayesian regression. All four diversity metrics—Shannon index, Simpson index, species richness, and Pielou’s evenness—revealed a significant positive correlation between native and alien diversity (Pearson’s *r* range: 0.112–0.155, all p<0.001), albeit with a weak correlation strength.

**Figure 12:**
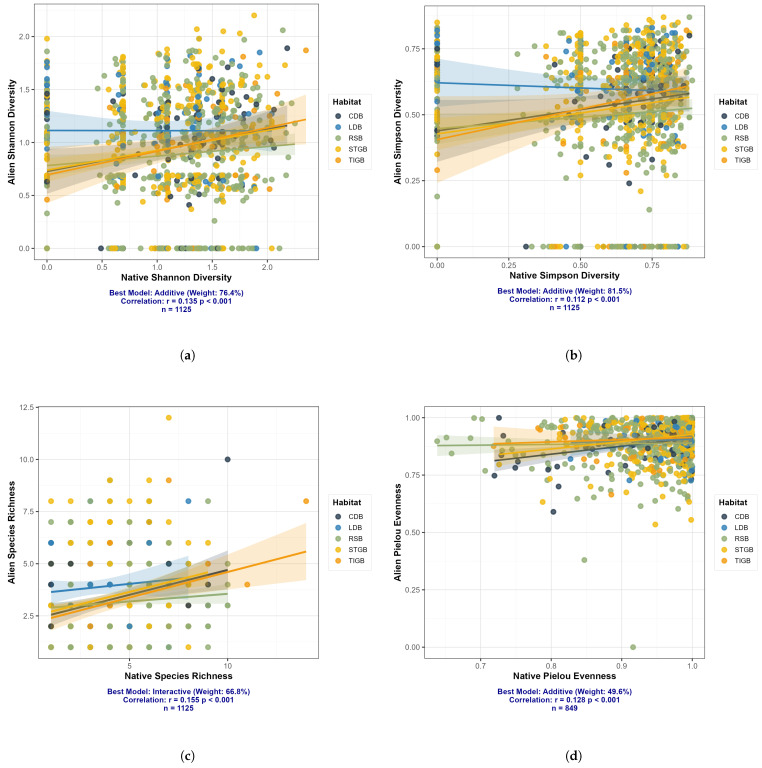
Multi-model Inference Analysis. (**a**) Shannon–Wiener Index. (**b**) Simpson’s Diversity Index. (**c**) Species Richness. (**d**) Pielou’s Evenness Index.

Model comparison analysis indicated that the model structure with the highest explanatory power varied with diversity metrics. For Shannon and Simpson diversity, the additive model (native diversity + habitat type) received the strongest support, with Akaike weights of 76.4% and 81.5% (Figure 12), respectively. Under this model, native diversity exerted a consistent positive effect on alien diversity (standardized coefficient β=0.132 and 0.112, respectively, both p<0.001). For species richness, the interaction model (native diversity × habitat type) was the top-supported model (weight =66.8%), yet it competed with the additive model (weight =33.2%) with a ΔAICc value of 1.40. This suggests that the relationship might vary across habitats, though the evidence remains inconclusive. The best-fit model for Pielou’s evenness was also the additive model (weight =49.6%), but its support was very close to that of the interaction model (weight =42.0%), indicating high model uncertainty.

The results of model averaging showed that, after accounting for the uncertainty of all candidate models, native diversity (richness_native, shannon_native, simpson_native, pielou_native) exerted a stable positive effect on all four alien diversity metrics (all full-model average coefficients were positive). Meanwhile, the effect of habitat type (with “HBL” as the reference category) was also evident; for instance, in the Shannon diversity model, the “LDB” habitat exhibited a significant positive effect. Collectively, these findings demonstrate that an increase in native plant diversity tends to be accompanied by a rise in alien plant diversity, and this relationship is prevalent across different habitats. However, the specific pattern (i.e., the presence or absence of interaction effects) differs across various dimensions of diversity.

## 3. Discussion

### 3.1. Composition Characteristics of Species Diversity of Exotic Spontaneous Plants in Road Green Spaces

Urbanization has profoundly shaped the family and genus composition of exotic spontaneous plants in road green spaces. This is manifested in the community being dominated by a few dominant families, while species richness is mainly contributed by a large number of oligotypic families and monotypic families. Although the number of genera is very rich, the contribution of dominant genera to the total number of species is insufficient, indicating a high degree of plant species differentiation and complex composition in this area. This phenomenon is also a direct reflection of the heterogeneity of the urban environment, which is consistent with the research results of Tian Zhihui et al. [39,40]. Asteraceae is an important source of exotic spontaneous plants, which is related to their strong adaptability, large number of species, and better ability to adapt to harsh environments [41]. In terms of species origin, a quarter of the exotic spontaneous plants in road green spaces are escaped species, and most of them are tropical species, indicating that human preferences have an important impact on the composition of exotic spontaneous plants [42]. In the statistics of geographical component composition, it is found that the exotic spontaneous plants in road green spaces are dominated by cosmopolitan species, which is significantly different from the pattern where tropical component families are dominant in southern cities [22]. This phenomenon occurs because, restricted by the geographical conditions of cold regions, the low-temperature environment screens out species with strong adaptability and prominent diffusion ability, and a large number of random immigrations lead to cosmopolitan species occupying an obvious dominant position.

There are significant differences in niche structure between spontaneous native and exotic plant communities. The spontaneous exotic community is strongly dominated by a single dominant species, Setaria viridis, and approximately 17.2% of spontaneous exotic species exhibit niche breadth exceeding random expectations, indicating that exotic plants may possess broader resource utilization capabilities or stronger environmental adaptability. This pattern may be related to the niche release of spontaneous exotic plants under frequent human disturbances, enabling them to gain an advantage in resource competition. The dominant exotic species show high niche overlap, suggesting that these species may have highly similar resource requirements or microhabitat preferences, and may even co-invade through interspecific facilitation. In contrast, the niche overlap network of native species is more dispersed, reflecting niche differentiation formed during long-term coexistence, which may contribute to maintaining the stability of spontaneous native communities. The above results support the hypothesis that “spontaneous exotic species can establish dominance through niche expansion and high-intensity niche overlap”. This is highly consistent with the findings of Zhang et al. that Erigeron annuus, an invasive plant species, exhibits the widest niche breadth, dominates the community, and mostly presents a competitive relationship with its main associated plant species [43]. Future studies can combine functional traits and long-term dynamic data to further reveal the causal relationship between niche structure and invasion success.

### 3.2. Adaptation Strategies of Exotic Spontaneous Plants Across Different Habitat Types and Urbanization Levels

There were no significant differences in the α-diversity of spontaneous exotic plants across different habitats [44,45], whereas the β-diversity was extremely high with a very low explanatory power, and this variation was almost entirely contributed by species turnover. This indicates that, although various habitats sustain a similar level of species richness of spontaneous exotic plants, their community compositions are heterogeneous, presenting a pattern of ‘similar diversity level yet distinct species composition’.

The lack of significant differences in α-diversity across habitats may reflect that spontaneous exotic plants are able to find exploitable niches or resource opportunities in all the habitats defined in this study, or indicate that the colonization of exotic plants in the study area has reached a certain state of ‘saturation’. In addition, anthropogenic disturbance, habitat fragmentation, or the reduction in dispersal limitation may have facilitated the establishment of spontaneous exotic species populations across different habitats, thereby homogenizing the diversity levels. The combined results of PERMANOVA and β-diversity partitioning analysis confirm that the differences in species composition across habitats, albeit limited, are statistically significant, and these differences are almost entirely derived from species turnover [46]. The assembly of spontaneous exotic plant communities may thus be more dependent on habitat-specific species replacement rather than the overall increase or decrease in species number. This implies that even if different habitats harbor a similar number of species, the differences in their species composition may still lead to variations in ecosystem functions [42].

Habitats such as road verges, median strips, and traffic islands exhibit differences in terms of width, soil volume, moisture conditions, intensity of anthropogenic trampling and mowing, and the impacts of vehicle exhaust and snow-melting agents. The heterogeneity of these physical and disturbance conditions constitutes an “environmental filter” [47] that selects for species with distinct tolerances and ecological strategies. For instance, drought-tolerant and compaction-resistant species may dominate the central median strips, whereas shade-tolerant species or those capable of withstanding intermittent waterlogging tend to concentrate in road verges or street tree green belts. This habitat-specific filtering mechanism may account for the high rate of species turnover alongside the convergence of overall species richness.

At the urban gradient scale, although a species composition pattern with high turnover was maintained among different habitat types, the variation in plant communities along the macro-spatial gradient from urban center to outer suburbs was extremely limited. Specifically, α-diversity only exhibited a weak statistical gradient with limited practical significance, while β-diversity failed to form a significant spatial differentiation pattern. This contrast may indicate that the filtering effect of local habitat characteristics has a stronger influence than the macro-scale urbanization gradient effect.

Principal Coordinates Analysis (PCoA) revealed that community composition did not exhibit differentiation along the urban gradient. This finding stands in contrast to the high species turnover rate observed among different habitat types (significant in PERMANOVA). This discrepancy may be attributed to the dominance of local habitat filtering over the urban gradient effect: specifically, the plant community in a traffic island in the urban center may differ substantially from that of the adjacent roadside community yet be quite similar to the plant community in a traffic island in the outer suburbs.

The conclusions of this study are based on a specific city, and the length and intensity of its urban gradient (e.g., the historical development of the urban center, suburban development modes) may affect the generalizability of the results.The low explanatory power of the “region type” indicator suggests that future studies should quantitatively identify specific local driving factors (e.g., soil physicochemical properties, precise disturbance frequency, surrounding land use types) [48,49] instead of relying on macro-scale classification schemes. Future research can combine functional trait and phylogenetic analyses to further test the hypothesis that habitats of the same type across different regions filter out plant communities with similar functional characteristics.

### 3.3. Diversity and Interspecific Relationships Between Exotic and Native Spontaneous Plants in Road Green Spaces

This study reveals that the interspecific associations among dominant spontaneous plant species in the road green spaces of Changchun exhibit a pattern of ‘overall significant positive correlation, but predominantly weak pairwise interactions’ [50,51]. The overall variance ratio indicates a positive correlation, suggesting a tendency for species coexistence at the landscape scale. However, correlation analyses of specific species pairs show that up to 100% of species pairs exhibit weak correlations (|r|≤0.3), with ecologically significant associations being extremely rare. This widespread pattern of weak correlations is distinctly different from the characteristics of natural ecosystems and represents a typical outcome of urban artificial habitats [52]. The formation of this pattern can be attributed to frequent and intense anthropogenic disturbances: routine management practices such as mowing, weeding, and trampling continually reset community succession processes, hindering the establishment and development of stable long-term interspecific relationships. It is also related to the significant and unstable heterogeneity of road habitats in terms of soil properties, moisture conditions, and pollution levels. In such environments, species distribution is more constrained by short-term tolerance and responses to heterogeneous habitats rather than fixed biotic interactions. Among these species, those capable of natural colonization and becoming dominant typically possess strong dispersal abilities, fast growth rates, and short life cycles, characteristics associated with broad ecological niches and an opportunistic survival strategy.

Combined with previously observed high species turnover rates among habitats and weak urban macro-gradient effects, the weak biotic interactions can be integrated into a continuous framework of “environmental filtering → random assembly → loose coexistence.” Within this framework, environmental filtering primarily operates between different habitat types, while random processes and weak biotic interactions dominate the specific species combinations within each habitat type. The current study deduces interspecific associations from coexistence data; future research should incorporate controlled experiments to verify the nature and strength of these interactions. Additionally, further analysis of the distribution of species functional traits is needed to clarify whether this “weak association” corresponds to high functional similarity (niche overlap) or significant trait differences (niche differentiation) among species.

Furthermore, by integrating multi-model inference and Bayesian analysis, a widespread but weak positive correlation was found between the diversity of spontaneous native and exotic plants in urban road green spaces. This finding contradicts the traditional view of the diversity-invasibility hypothesis—which posits that highly diverse communities tend to inhibit the natural colonization of exotic species—and instead supports the co-variation hypothesis or habitat filtering mechanism. That is, specific environmental factors such as resource enrichment and disturbance can simultaneously promote the diversity of both native and spontaneous exotic plants. This conclusion aligns with the findings of indicating that under different environmental conditions, mutualistic interactions among species can determine the success or failure of biological invasions.

The weak positive correlation between spontaneous native and exotic plant diversity likely stems from their coordinated response to habitat heterogeneity. As mentioned earlier, exotic spontaneous herbaceous plants exhibit high turnover rates across different habitat types (e.g., roadside verges, central medians). Habitat patches with complex microenvironments, diverse resource types, or moderate disturbance intensities can provide opportunities for the colonization and coexistence of both spontaneous native and exotic plants. Therefore, the observed positive correlation is not a result of direct causal interactions between species but rather a parallel response of both groups to highly suitable or high-capacity habitats [53].

The widespread pattern of weak interspecific associations identified in this study provides crucial ecological context for the aforementioned positive correlation. In habitats dominated by high environmental stress (e.g., anthropogenic disturbance, soil constraints) and random dispersal, intense competitive exclusion among successfully established species is diminished. This loose community structure reduces the biotic resistance faced by new colonizers (including exotic species), allowing native and spontaneous exotic plants to share niche spaces shaped by environmental heterogeneity without strong mutual exclusion.

Analysis based on the Shannon and Simpson indices (reflecting species richness and evenness) shows that additive models dominate, indicating that the positive effect of spontaneous native plant diversity is relatively consistent across different habitats. This implies that community structures or resource states capable of maintaining high richness and evenness of spontaneous native plants inherently provide invasion opportunities or utilizable resource spectra for exotic species. In the analysis of species richness, interactive models received some support, suggesting that the slope of the spontaneous native–exotic species richness correlation may vary with habitat type. For example, in habitats with high resource variability and intense disturbance (e.g., central medians), spontaneous native plants may struggle to form effective competitive barriers, resulting in a neutral or positive correlation between spontaneous native and exotic species richness. Conversely, in habitats with relatively stable resources and complex structures (e.g., wide road green belts), native communities may exert a weak inhibitory effect on exotic species, flattening the slope of the positive correlation.

All core findings of this study can be integrated into a unified conceptual model describing the plant community assembly process in urban road ecosystems: At the landscape scale, habitat filtering dominates. At the patch scale, habitat heterogeneity and random dispersal jointly shape diversity levels. Within specific habitats, local environmental heterogeneity and stochastic seed dispersal events collectively determine the total number of species a patch can accommodate (α-diversity). Highly heterogeneous patches can simultaneously support more native and exotic spontaneous species, resulting in a weak positive correlation between their diversity.

Within communities, loose coexistence and weak interactions are characteristic. Under high-frequency disturbances and environmental stress, successfully coexisting species form a widespread pattern of weak associations, with intense competition or mutualism being extremely rare. Community structure exhibits a high degree of stochasticity and plasticity.

It should be noted that the causal mechanisms underlying the positive correlations revealed by this observational study require validation through controlled experiments such as seed addition or species removal. Future research should directly quantify key environmental factors like soil nutrients and physical stress intensity, and integrate species functional trait spectra to more precisely elucidate the mechanism chain of environmental filtering → species coexistence.

## 4. Materials and Methods

### 4.1. Overview of the Study Area

Changchun City (43°10′–45°15′ N) is situated in Northeast China and experiences a temperate continental monsoon climate. Based on the 1991–2020 climate normals, the city has an annual mean temperature of 6.6 °C and receives an average annual precipitation of 592.7 mm. Seasonal contrasts are pronounced: the warmest month (July) averages 21.9 °C, while the coldest month (January) averages –12.0 °C. Spring is characterized by strong winds, with maximum speeds occasionally reaching 30 m/s, whereas autumn typically sees milder wind conditions.

In the regional context, families such as Asteraceae, Amaranthaceae, Poaceae, Fabaceae, and Solanaceae are reported to be among the main families of exotic invasive plants in Jilin Province. At the urban scale, according to the Changchun Statistical Yearbook 2022, roads occupy approximately 12% of the built-up area, with both road-green coverage and road density having shown increasing trends in recent years.

### 4.2. Research Methods

#### 4.2.1. Plot Setup

**Figure 13:**
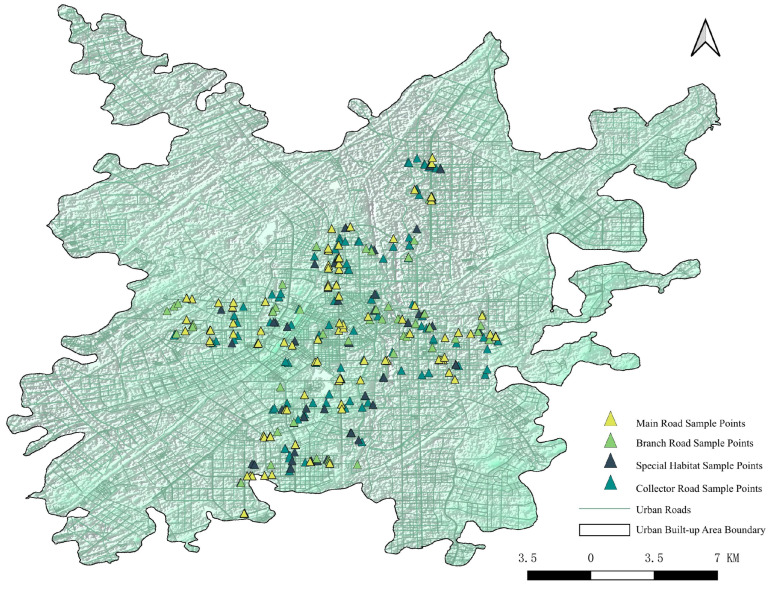
Distribution Map of Surveyed Sampling Sites.

In ArcGIS, a cross-shaped transect with a width of 5 km was drawn with Changchun’s city center (People’s Square) as the origin, ensuring coverage of most road distribution areas within the built-up area (Figure 13). The species names of street trees within the transect were identified via Baidu Maps Street View and field surveys. Dominant tree species were selected to set up research sampling points, with the number of quadrats for different dominant tree species kept approximately equal. During the survey, centered on each selected sampling point, one 20 m × 20 m arbor quadrat, four 5 m × 5 m shrub quadrats, and five 1 m × 1 m herb quadrat were laid out. The length and width of the quadrats were set according to the crown width of the arbor trees, with the requirement that crown width × length = 400 m^2^. Information was recorded for spontaneous plants in 1268 quadrats across 269 sampling sites, including their names, cultivation types, height, coverage, diffusion types, as well as the management status, health conditions of street trees, and road-related information. Clear photographs were taken of the habitats, surrounding environments, overall road scenes, and each layer of the quadrats.

#### 4.2.2. Habitat Type Classification

**Figure 14:**
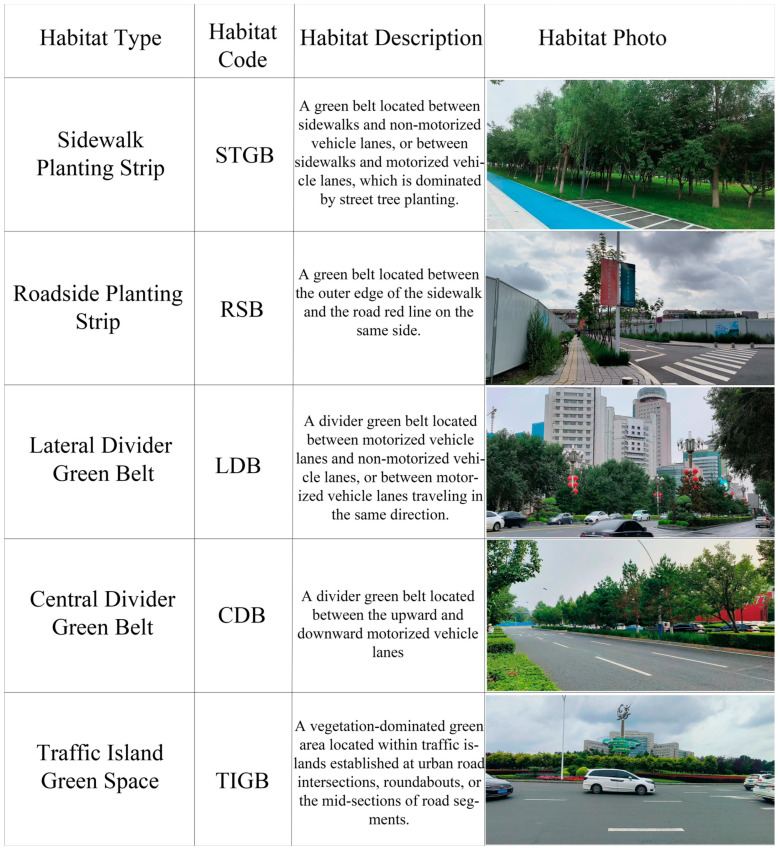
Habitat Classification Standards.

In accordance with the Standard for Classification of Urban Green Space (CJJT 85-2017), the urban road green spaces in Changchun City were divided into the following 5 categories based on the different positions of green belts within the road cross-section (Figure 14).

#### 4.2.3. Classification of Urbanization Level and Disturbance Intensity

Based on the 10 m resolution Landsat series satellite images from 2023, this study used ENVI 5.6 and ArcGIS 10.7 to interpret the land cover of the study area into five types: urban construction land, water bodies, vegetation, cultivated land, and bare land. The maximum distance from each sampling point to the urban boundary was calculated to characterize the urbanization level, and the equal interval method was used to divide the study area into three urbanization level gradients: outer suburbs, inner suburbs, and city center.

### 4.3. Data Processing

(1) This study quantified and compared the ecological strategies of native and exotic plant communities using three complementary niche metrics. First, we calculated the overall importance value for each species by integrating its relative frequency and abundance across all plots. Second, the niche breadth of each species was assessed using Levin’s standardized index, which measures the uniformity of a species’ distribution across environmental gradients (ranging from 0, specialist, to 1, generalist). Third, pairwise niche overlap among species was quantified using Pianka’s index, ranging from 0 (no overlap) to 1 (complete overlap). To control for the influence of dominant species and reduce computational complexity, analyses of niche breadth and overlap were focused on the top 30 species ranked by overall importance. To determine whether the observed niche patterns differed from random expectations, we performed null model analyses. We used a randomization algorithm that shuffled species occurrence and abundance data among plots while preserving the matrix structure. Significance was tested with 999 permutations, and resulting p-values were adjusted using the False Discovery Rate (FDR) correction. All statistical analyses and visualizations were performed in R version 4.5.2. The average value of the experimental data is calculated using the following formula:

(1) $${\mathrm{IV}}_{i}=\frac{{\mathrm{RF}}_{i}+{\mathrm{RC}}_{i}}{2}$$

In the formula, ${\mathrm{IV}}_{i}$ represents the importance value of the *i*-th species; ${\mathrm{RF}}_{i}$ represents the relative frequency of the *i*-th species, and ${\mathrm{RC}}_{i}$ represents the relative coverage of the *i*-th species.

- Relative Frequency (RF) = Frequency of a certain species in the quadrat/Sum of frequencies of all species
- Relative Coverage (RC) = Coverage of a certain species in the quadrat/Sum of coverages of all species

(2) Calculation of α-diversity, including the Richness index (R), Shannon–Weiner diversity index ($H^{\prime }$), Pielou evenness index (*J*), and Simpson dominance index (*D*). The calculation formulas are as follows:

(2) R=S

In the formula, *S* denotes the number of species in the quadrat.

(3) $$H^{\prime }=-\sum_{i=1}^{S}p_{i}\ln p_{i}$$

where:

- $H^{\prime }$: Shannon-Weiner diversity index
- *S*: total number of species
- $p_{i}$: relative abundance of the *i*-th species, $p_{i}=\frac{n_{i}}{N}$
- $n_{i}$: number of individuals of species *i*
- *N*: total number of individuals, $N=\sum_{i=1}^{S}n_{i}$

The Pielou evenness index (*J*) is calculated as:

(4) $$J=\frac{H^{\prime }}{H_{\max}}=\frac{H^{\prime }}{\ln S}$$

where:

- *J*: Pielou evenness index (ranges from 0 to 1)
- $H^{\prime }$: observed Shannon-Weiner diversity index
- $H_{\max}$: maximum possible diversity, $H_{\max}=\ln S$
- *S*: total number of species

The Simpson dominance index (*D*) is calculated as:

(5) $$D=\sum_{i=1}^{S}p_{i}^{2}$$

where:

- *D*: Simpson dominance index (ranges from 0 to 1)
- $p_{i}$: relative abundance of the *i*-th species, $p_{i}=\frac{n_{i}}{N}$
- *S*: total number of species

(3) The probability of species presence across habitat types was modeled using generalized linear models with a logit link function:

(6) $$\begin{array}{cc} \log\left(\frac{p_{ij}}{1-p_{ij}}\right) & =\beta_{0}+\beta_{1}\cdot {\mathrm{CityCenter}}_{j}+\beta_{2}\cdot {\mathrm{MiddleSuburbs}}_{j}\\ \; & \;+\beta_{3}\cdot {\mathrm{OuterSuburbs}}_{j}+\epsilon_{ij} \end{array}$$

where:

- $p_{ij}$ is the probability of presence (either alien or native species) in plot *i* within habitat type *j*
- $\beta_{0}$ is the intercept (baseline log-odds when all predictors are 0)
- $\beta_{1}$, $\beta_{2}$, $\beta_{3}$ are coefficients for habitat types (with reference level encoded in the model matrix)
- ${\mathrm{CityCenter}}_{j}$, ${\mathrm{MiddleSuburbs}}_{j}$, ${\mathrm{OuterSuburbs}}_{j}$ are indicator variables for the three habitat types
- $\epsilon_{ij}$ is the error term

Separate models were fitted for alien and native species presence.

### 4.4. α-Diversity Differences Among Habitats (Linear Models)

To compare diversity indices among habitat types, linear models were applied after appropriate transformations to meet normality assumptions:

(7) $$\begin{array}{cc} Y_{ijk}^{\prime } & =\mu +\alpha_{j}+\epsilon_{ijk} \end{array}$$

(8) $$\begin{array}{cc} \epsilon_{ijk} & ∼N(0,\sigma^{2}) \end{array}$$

where:

- $Y_{ijk}^{\prime }$ is the transformed diversity index value for:
  - Species richness: $Y^{\prime }=\log\left(Y+1\right)$
  - Shannon index: $Y^{\prime }=\sqrt{Y+0.01}$
  - Pielou’s evenness: $Y^{\prime }=Y$ (no transformation, range 0–1)
  - Simpson’s index: $Y^{\prime }=Y$ (no transformation, range 0–1)
- μ is the overall mean
- $\alpha_{j}$ is the fixed effect of habitat type *j* (j=1,2,3)
- $\epsilon_{ijk}$ is the residual error, assumed normally distributed with mean 0 and variance $\sigma^{2}$

When the overall F-test from Type III ANOVA was significant (p<0.05), **Tukey’s Honestly Significant Difference (HSD)** post hoc test was conducted for all pairwise comparisons between habitat types.

### 4.5. Diversity Trends Along Urbanization Gradient (Linear Regression)

To test for linear trends in diversity indices along the urbanization gradient, simple linear regression models were fitted:

(9) $$\begin{array}{cc} Y_{ik}^{\prime } & =\beta_{0}+\beta_{1}\cdot X+\epsilon_{ik} \end{array}$$

where:

- $Y_{ik}^{\prime }$ is the transformed diversity index (same transformations as above)
- *X* is the numerical urbanization gradient: City Center = 1, Middle Suburbs = 2, Outer Suburbs = 3
- $\beta_{0}$ is the intercept
- $\beta_{1}$ is the slope coefficient representing the linear trend
- $\epsilon_{ik}$ is the error term

## 5. Conclusions

This study clarifies the diversity distribution patterns and adaptive mechanisms of spontaneous plant communities in road greenbelts of Changchun City; the key conclusions are as follows. At the family and genus levels, exotic spontaneous plants are characterized by a composition dominated by dominant families and enriched with oligotypic families, and their niche structure features the predominance of single dominant species, broad niche breadth, and high niche overlap, which reflects their adaptive expansion strategies under frequent anthropogenic disturbances. There are no significant differences in the α-diversity of exotic spontaneous plants across different habitats, whereas extremely high species turnover rates are observed among habitat types, indicating that the filtering effect of local habitats plays a dominant role, while the impact of macro-urbanization gradients is negligible. In addition, a weak positive correlation exists between the diversity of exotic and native spontaneous plants, and a prevalent pattern of weak interspecific associations is exhibited throughout the entire community. Collectively, these findings support a unified conceptual model of **“environmental filtering → stochastic assembly → loose coexistence”**; specifically, at the landscape scale, species are filtered by habitat heterogeneity, within habitat patches, community assembly is dominated by stochastic dispersal and weak biotic interactions, and ultimately, this process gives rise to urban artificial plant communities with a loose structure and high plasticity.

## Figures and Tables

**Table 1:** Genus Composition of Exotic Spontaneous Plants.

	Genus Type				
	Monotypic Genus	Ditypic Genus	Trittypic Genus	Tetratypic Genus	Pentatypic Genus
Genus/Species	127/127	29/58	8/24	4/16	2/10
Percentage (%)					
Genus proportion	74.7	17.1	4.7	2.4	1.2
Species proportion	54.0	24.7	10.2	6.8	4.3

Monotypic genus: genera with one species; Ditypic genus: genera with two species; Trittypic genus: genera with three species; Tetratypic genus: genera with four species; Pentatypic genus: genera with five species.

## Data Availability

The data presented in this study are openly available in 13 November 2025. Figshare at https://doi.org/10.6084/m9.figshare.30606755.v1.

## Appendix A

The information about native plants and their codes involved in the interspecific association analysis in the article is shown in the table below.

**Table A1:** Table of Interspecific Association Species.

Species Code	Latin Name
sp11	Echinochloa crus-galli
sp19	Polygonum aviculare
sp27	Potentilla supina
sp28	Plantago asiatica
sp35	Cirsium arvense var. integrifolium
sp54	Stellaria media
sp55	Amaranthus retroflexus
sp62	Setaria viridis
sp77	Youngia japonica
sp94	Sonchus wightianus
sp98	Sonchus oleraceus
sp105	Chenopodium album
sp115	Solanum nigrum
sp120	Cynanchum rostellatum
sp122	Portulaca oleracea
sp124	Digitaria sanguinalis
sp137	Galinsoga parviflora
sp148	Taraxacum mongolicum
sp189	Acalypha australis
sp211	Polygonum plebeium
sp221	Erigeron canadensis
sp232	Commelina communis
sp238	Glycine soja
sp243	Lactuca serriola
sp254	Ulmus pumila
sp262	Viola prionantha
sp274	Ixeris chinensis
